# Rational Design of P450 aMOx for Improving Anti-Markovnikov Selectivity Based on the “Butterfly” Model

**DOI:** 10.3389/fmolb.2022.888721

**Published:** 2022-05-23

**Authors:** Yue Pan, Jinxiao Bao, Xingyi Zhang, Hui Ni, Yue Zhao, Fengdong Zhi, Bohuan Fang, Xiao He, John Z. H. Zhang, Lujia Zhang

**Affiliations:** ^1^ Shanghai Engineering Research Center of Molecular Therapeutics and New Drug Development, Shanghai Key Laboratory of Green Chemistry and Chemical Process, School of Chemistry and Molecular Engineering, East China Normal University, Shanghai, China; ^2^ College of Food and Biological Engineering, Jimei University, Xiamen, China; ^3^ NYU-ECNU Center for Computational Chemistry at NYU Shanghai, Shanghai, China; ^4^ Department of Chemistry, New York University, New York, NY, United States

**Keywords:** anti-Markovnikov (AM) oxidation, product selectivity, rational design, molecular dynamic simulations, experimental alanine scan, “butterfly” model

## Abstract

Aromatic aldehydes are important industrial raw materials mainly synthesized by anti-Markovnikov (AM) oxidation of corresponding aromatic olefins. The AM product selectivity remains a big challenge. P450 aMOx is the first reported enzyme that could catalyze AM oxidation of aromatic olefins. Here, we reported a rational design strategy based on the “butterfly” model of the active site of P450 aMOx. Constrained molecular dynamic simulations and a binding energy analysis of key residuals combined with an experimental alanine scan were applied. As a result, the mutant A275G showed high AM selectivity of >99%. The results also proved that the “butterfly” model is an effective design strategy for enzymes.

## Introduction

Aromatic aldehydes are important intermediate compounds in the industry and have a wide range of applications in chemical, pharmaceutical, and daily chemical fields (Dubrovskiy et al., 2018). The direct synthesis of aromatic aldehydes from aromatic olefins *via* the anti-Markovnikov pathway greatly simplifies the synthesis procedure in industrial production (Dong et al., 2015; Wu et al., 2019). However, due to the complex mechanism, the AM selectivity is still a challenge. Currently, metal organics are mainly used as catalysts for the oxidation of olefins to synthesize such substances (Beller et al., 2004). Although some achievements have been made in related research studies (Chen et al., 2011; Nguyen et al., 2019), the complexity of the structural modification of metal-derivative catalysts still remains to be addressed.

Compared with metal-organic catalysts, biocatalysts have the advantages of abundant sources, high reactivity, and environmental friendliness (Musa and Phillips, 2011; Sheldon and Woodley, 2018). Among biocatalysts, the widely distributed NAD(P)H-dependent P450 mono-oxygenases can be used to catalyze hydroxylation, epoxidation, and nitration (Barry et al., 2012; Guengerich and Munro, 2013; Dodani et al., 2016; Girvan and Munro, 2016). P450s have become a hotspot for biocatalyst research, owing to the diversity of their catalytic reactions (Sono et al., 1996). In 2017, Arnold et al. reported that P450 aMOx, a Class IV self-sufficient cytochrome P450 (Munro et al., 2007), from directed evolution could catalyze the oxidation of aromatic olefin styrene to phenylethylaldehyde with a high selectivity at 81% (Hammer et al., 2017). Many achievements have been realized *via* direct evolution (Reetz et al., 2004; Kan et al., 2016; Buller et al., 2018), and the mutations from directed evolution consist of a mutagenesis library for further study (Acevedo-Rocha et al., 2018). However, directed evolution is laborious, time-consuming, costly (Yang et al., 2019), and lack a regular enzyme design modification method. Hence, this study used a combination of rational designs and experimental methods to redesign aMOx in order to further improve the AM selectivity for the catalysis of aromatic compounds.

## Materials and Methods

### Cloning, Expression, and Purification

The complete aMOx gene sequence was obtained from a previous report (Hammer et al., 2017). Synthesis of gene sequence, mutant primers, and sequencing were assigned to Shanghai Sunny Biotechnology Co. (Shanghai, China). The pET 22b (Invitrogen, Carlsbad, CA, United States) was used as the vector, and the target gene was located between *Nde I* and *Hind III* enzymatic sites with a C-terminal 6*His Tag. The mutants were constructed by using a seamless cloning kit from Beyotime Biotechnology (Shanghai, China). *Escherichia coli* DH5α (Weidi Biotechnology Co., Ltd., Shanghai, China) was used as the vector cloning host, and *E. coli* BL21 (DE3) (Weidi Biotechnology Co., Ltd., Shanghai, China) was used as the expression host. All chemicals applied were of AR grade.

Bacteria harboring both aMOx and its mutants were cultivated and expressed in an LB medium containing 100 µg/ml ampicillin at 37°C and allowed for shaking at 250 rpm for 3 h, for protein expression. When the OD_600_ value of the bacterial solution was between 0.6 and 0.8, the final concentrations of 0.1 mM IPTG and 0.5 mM 5-aminolevulinic acid (5-ALA) were added. The system was induced at 20°C, 250 rpm for 12 h. The system was centrifuged at 8,000 rpm for 5 min to collect the bacteria. The pellet was treated with a crushing solution at the ratio of 10 ml of crushing solution (0.1 M PBS, 150 mM NaCl, and 10% glycerol; pH 8.0) to 1 g of wet bacterium and re-suspended on ice with ultrasonic crushing for 30 min. The crushing solution was centrifuged at 20,000 g for 15 min to take the supernatant. The target protein was purified using 5 ml Ni-NTA and AKTA PURE system (Cytiva, Boston, MA, United States). The target proteins were eluted with 0.1 M PBS; 150 mM NaCl; 10% glycerol; and 20, 50, 200, and 500 mM imidazole; each gradient elution volume had five columns as the volume. Target proteins of aMOx and mutants were eluted by 200 mM imidazole. The buffer was replaced by using 30kD ultrafiltration tubes with 0.1 M PBS, 150 mM NaCl, and 10% glycerol, at pH 8.0, frozen in liquid nitrogen and stored in the refrigerator at −80°C.

### Homology Modeling and Molecular Docking

Because the substrate selectivity of P450 is exclusively related to its oxidant domain, this study specifically targeted the oxidative structural domain (450 aa) of aMOx for structural analysis. The Protein BLAST module of the NCBI database was used for homologous sequence alignment of aMOx amino acid sequences, and CYP116B5 (Ciaramella et al., 2019) (PDB ID: 6RO8) with 82% sequence identity was used as the template. The homology modeling module of Discovery Studio was used for homology modeling of the oxidant domain of aMOx.

The Glide module of Maestro was used for molecular docking of the substrate styrene to aMOx to obtain possible structures of the substrate binding complex. The docking was centered on the heme oxygen, the grid size was set to 20 Å, and the distance between the olefin reaction site of the constrained styrene and the heme oxygen was 4 Å for constrained docking. The structure with the highest score was selected for subsequent studies.

### Specific Activity Test for aMOx and Variants

The specific activity of aMOx and mutants was determined by evaluating the consumption of NADPH (Liu et al., 2021). The Bradford (1976) method was used to determine the protein concentration using a Bradford protein quantification kit (YEASEN Biotechnology Co., Ltd., Shanghai, China). The reaction conditions were as follows: 0.1 μM purified enzyme, 0.1 mM styrene, and 0.2 mM NADPH in 25 mM Tris-HCl, at pH 8.0. The reaction volume was 200 μl. The reaction system was shaken for 30 min at 25°C. Then, a Multiskan Sky microplate spectrophotometer (Thermo Scientific, Waltham, MA, United States) was used to detect the change of absorbance value at 340 nm within 30 min.

### High-Throughput Screening of aMOx Variants for Anti-Markovnikov Selectivity

**SCHEME 1:**
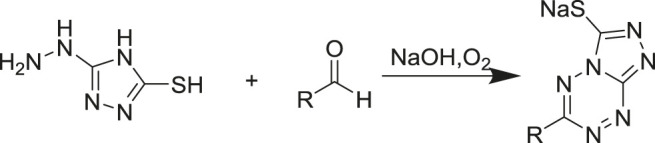
Reaction mechanism of Purpald.

Purpald (Hopps, 2000) (4-amino-5-hydrazino-1, 2, 4-triazole-3-thiol) is a class of chromogenic agents that reacts specifically with aldehydes to form purple complexes, which are complexation products with aldehydes that have a characteristic absorption at 528 nm (Scheme 1). In this study, Purpald was used as a derivatization reagent for the quantification of aMOx and the AM selectivity of the mutant based on this property. Purpald showed good linearity for 0–5 mM of phenylethylaldehyde. The reaction conditions were as follows: 100 μl lysis enzyme (approximately 1 μM aMOx), 5 mM substrate, 10 mM NADPH, and 25 mM Tris-HCl at pH 8.0 to make up the reaction volume of 200 μl, 20°C, 900 rpm for 2 h. The reaction was terminated by adding 50 μl 31 mM Purpald and incubated for 30 min at 25°C, and the absorbance value at 538 nm was measured.

### Molecular Dynamics Simulations and Molecular Mechanics Generalized Born Surface Area Analysis

Molecular dynamics simulations (MDs) were performed with Amber18 and AmberTools19 software applications. The ff14SB force field was used for protein, the gaff force field and the AM1-BCC charge model were used for organic ligands, the parameters provided by Shaik et al. (2010) and Shahrokh et al.( 2012) were used for the Cpd I group, and the TIP3P waters and the Li/Merz monovalent ion parameters were also used. The water box was set to be truncated ortho-octahedrally, and the minimum distance between complex atoms and box boundaries was set to be 12.0 Å; Na^+^ and Cl^−^ ions were added according Shahrokh et al. (2012) to neutralize the system, and the ionic strength was made near 0.15 mol/L.

First, the initial system was minimized with the 5,000-step steepest descent method (SD), followed by the 5,000-step conjugate gradient method (CG) with harmonic Cartesian space restraints on all complex atoms; the restraint weight was set to be 10.0 kcal mol^−1^ Å^−2^. Next, the Cartesian space restraints were removed, and the whole system was further minimized with 10,000-step SD and 10,000-step CG. Subsequently, the system was progressively heated from 0 K to the target temperature (300.0 K) within 300 ps under NVT ensemble and then equilibrated to the target pressure (1.0 bar) within 1.0 ns under NPT ensemble. The Langevin thermostat with the collision frequency set as 2.0 ps^−1^ and the Monte Carlo barostat with the relaxation time set to 2.0 ps were used. Harmonic Cartesian space restraints were added on all complex atoms during heating and equilibration with the weight set to 5.0 and 2.0 kcal mol^−1^ Å^−2^, respectively. During the MDs, the SHAKE method was used to constraint bonds involving hydrogen atoms, and the time step was set to 2.0 fs; the PME method was used to calculate the long-range electrostatic energy, and the cutoff on the long-range van der Waals (VDW) energy was set to 10.0 Å.

To retain the substrate’s vinyl group near the oxygen of Cpd I, during the minimization, heating, and equilibration periods, the NMR type of distance restraints were added between the vinyl carbons and the Cpd I oxygen. When the actual distance was less than the target distance, the restraint weight was zero, whereas when the actual distance was larger than the target distance, the restraint weight was set to 3.0 kcal mol^−1^ Å^−2^.

To analyze the receptor residue binding energy contribution to the substrate when the substrate is close to the reaction center, the NMR distance restraint was added between position 1 of the substrate and the Cpd I oxygen with the target distance set to 3.0 Å. After the equilibration, the Cartesian space restraints were removed, and the system was produced with different random seeds five times with the NMR distance restraint kept. The simulation duration of each production was set to 100 ns, and snapshots were recorded every 100 ps to form the trajectory for further analysis. The molecular mechanics generalized born surface area (MMGBSA) calculations were performed on the last 800 frames (80 ns) of each trajectory, and the binding energy was decomposed to each receptor residue (except for the Cpd I group). During the calculation, the GBOBC model I (igb = 2) was used as the implicit solvent model, the interior dielectric constant was set to 3.0, the ionic strength was set to 0.1 mol/L, the LCPO method was used to calculate the solvent accessible surface area, and the surface tension was set to 0.0005. The corresponding values of all the five trajectories were averaged to get the final results.

To analyze the contact between the substrate vinyl carbons and the Cpd I oxygen of different protein mutants, the NMR distance restraints were added both on the position 1 carbon and position 2 carbon of the substrate to the Cpd I oxygen during minimization, heating, and equilibration with the target distance set to 3.5 Å. Then, the system was further equilibrated during 5 ns with only the NMR distance restraint kept and then equilibrated for 5 ns with no restraints. After these equilibrations, the accelerated molecular dynamics (aMD) were performed on these systems. The system’s averaged total potential energy and dihedral energy were calculated among the last 4 ns of the equilibrations. The aMD parameter: alphaD was set to be 0.7 times the number of the complex residues, and alphaP was set to be 0.2 times the number of system total atoms. Both the system’s total potential energy and the dihedral energy were boosted during the aMD calculation, and the boost energy on the dihedral energy term was set to be twice of alphaD. The time step was set to 1.5 fs during aMD, and the relative geometrical tolerance of SHAKE and the dsum_tol parameter of PME were both set to 0.000001. Snapshots were recorded every 7.5 ps to form the trajectory. Each system was calculated with different random seeds ten times, and the aMD duration of each production was set to 30 ns.

## Results and Discussion

### Computational Analysis of Multiple Rounds of aMOx-Directed Evolution and Establishment of the “Butterfly” Catalytic Model

**SCHEME 2:**
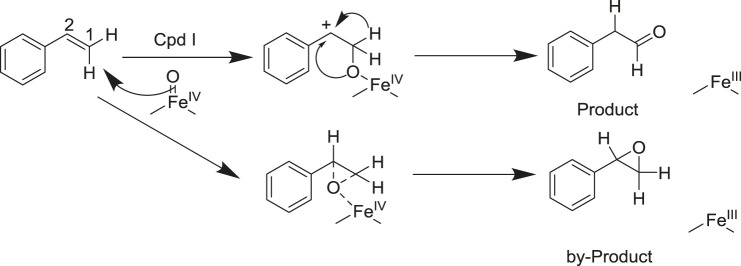
Reaction mechanism of the aMOx-catalyzed olefin Markovnikov oxidation reaction (supposed).

P450 aMOx is a self-sufficient P450 mono-oxygenase produced by 10 rounds of directed evolution of P450_LaMO_ (Yin et al., 2014). The predicted structure of P450 aMOx was determined by homology modeling (Figure 2A). Since the Cpd I is a highly active intermediate of the P450 aMOx catalyzing cycle (Rittle and Green, 2010; Krest et al., 2013), its homology-modeled structure was both Cpd I and the substrate styrene, through molecular docking (Figure 2B). A previous report (Hammer et al., 2017) has described the possible mechanism of the aMOx-catalyzed AM oxidation reaction as a 1, 2-σ migration rearrangement reaction (Bonneau et al., 1989; Kim et al., 2003). A possible reaction mechanism is as follows: the substrate approaches the oxygen atom on heme, the active center of the aMOx reaction; next, the oxygen atom attacks position 1 of the aromatic olefin substrate, and the olefin’s double bond opens to form a carbon positive ion at position 2; this process induces the hydrogen ion at position 1 and migrates to position 2; simultaneously, the double bond formed by heme iron and oxygen breaks; the reaction is completed after the oxygen atom forms a double bond with carbon at position1 (Scheme 2).

The reaction distance between the reaction site and electrophilic reagent in AM oxidation was 3.0–4.0 Å in our pre-MD simulations. The near attack configuration (NAC) angle of the nucleophilic reagent, which is C7/C8-O-Fe, affects the selectivity of the AM oxidation reaction (Feng et al., 2019) (Figure 2B). We first analyzed the results of directed evolution reported by Arnold et al. *via* MD simulations in order to investigate the effects of these factors on AM selectivity and to establish a catalytic model and an efficient strategy for aMOx-catalyzed AM reaction. Among the 10 rounds of the directed evolution results, round 2 yielded a mutant with the highest increase (from 45% to 55%) in AM selectivity. Round 8 had 76% AM selectivity, which differed from the reported final selectivity of 81% by only 5%. Therefore, we selected round 1 (P450_LaMO_), round 2, round 8, and aMOx for a molecular dynamics simulation analysis. Mutations of residues could alter the steric hindrance of the active pocket and affect the way the reaction substrate contacts the active site, which is considered as one of the common causes of altered reaction selectivity due to residue mutations (Chen et al., 2018; Wang et al., 2020). The connection between the carbon atom on the vinyl of the substrate and the oxygen atom in Cpd I was investigated by aMD, an enhanced sampling tool (Hamelberg et al., 2004).

Distances between the carbons at positions 1 (C8-O) and 2 (C7-O) and the oxygen on Cpd I, in the respective 10–30 ns aMD simulation trajectories of the aforementioned systems, as well as time-related variation in the angle made by these two carbons with oxygen and iron atoms in Cpd I, respectively, are shown in Supplementary Figures S1, S2.

**FIGURE 1:**
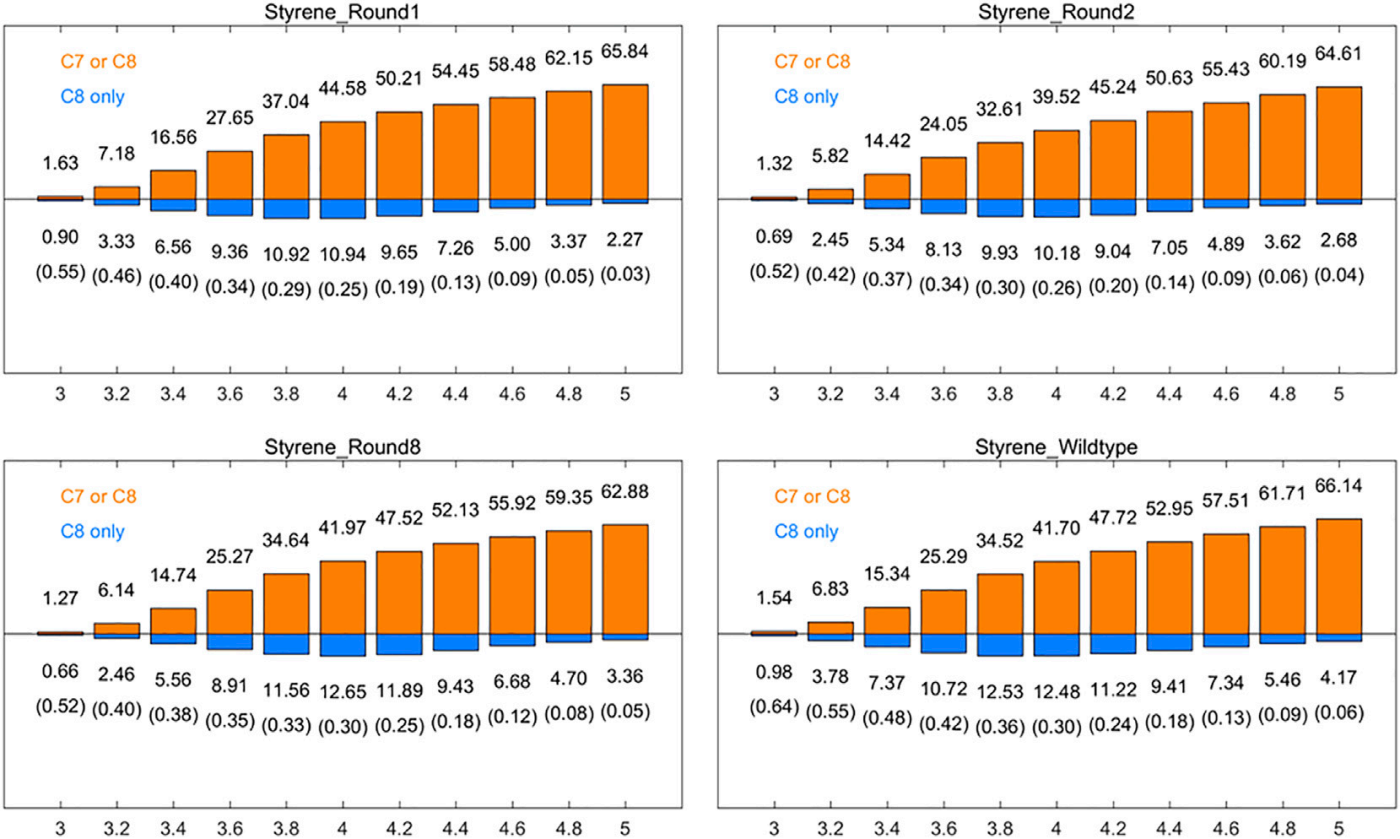
Percentages [p (C7 or C8), orange] of the frames that have at least one carbon (of styrene’s position 1 and position 2 carbons) within the given distance (x-axis, in Å) to the Cpd I oxygen among all the aMD frames, and the percentages [p (C8only), blue] of the frames where only position 1 carbon of styrene vinyl is within the given distance. The ratios of p (C8 only)/p (C7 or C8) are also listed in the brackets. All results are the averages of the corresponding ten 30 ns aMD trajectories.

It is supposed that spatial positional selectivity also lead to a significant influence in enzyme-catalyzed reactions. The frequency of reactive site 1 and non-reactive site 2 appearing within a 3–5 Å radius of the oxygen atom of Cpd I was further analyzed. We calculated the number of frames in which at least one of C7 and C8 of round 1, round 2, round 8, and aMOx has a distance to the oxygen on Cpd I smaller than a specific threshold as a percentage of the total number of frames of the corresponding trajectory [denoted as p (C7 or C8), Figure 1, orange bar], while the horizontal coordinates of the graph indicate the corresponding distance threshold. We also analyzed the percentage of the total number of frames in the substrate with the distance from the carbon at site 1 to the oxygen on Cpd I only less than this threshold [notated as p (C8 only)] at the corresponding threshold (Figure 1, blue bar). The corresponding ratio of p (C8 only) top (C7 or C8) is recorded in parentheses in Figure 1. We focused on the ratio of p (C8 only) to p (C7 or C8) at the reaction distance of 3.6–4.0 Å. The p (C8 only)/p (C7 or C8) values for round 1, round 2, round 8, and wild type are 0.34, 0.34, 0.35, and 0.42, respectively, when the distance threshold is 3.6 Å; when the distance threshold is 3.8 Å, the p (C8 only)/p (C7 or C8) values of round 1, round 2, round 8, and wild type are 0.29, 0.30, 0.33, and 0.36, respectively; when the distance threshold is 3.8 Å, the p (C8 only)/p (C7 or C8) values were 0.25, 0.26, 0.30, and 0.30, respectively. p (C8 only)/p (C7 or C8) trends for the three different thresholds were consistent with the trends of the inverse martensite rule product selectivity.

Inspired by the results of the distance analysis, we analyzed the NAC angle (C7/C8-O-Fe) *via* the same method. We first considered the angle as the standard and then calculated the number of frames in which at least one of the NAC angles is more than the given angle [denoted as p (C7 or C8)] and the number of frames in which only C8-O-Fe is more than the given angle [denoted as p (C8only)] of all track frames that meet the angle standard under different distance indexes. The consistency between p (C8 only)/p (C7 or C8) of different systems and their product selectivity is the same when the distance is 3.6, 3.8, 4.0, 4.2, and 4.4 Å, and 90°, 105°, 120°, and 135° are used as angle standards (Supplementary Figures S3–S8). The Spearman rank correlation was better with the angle standard of 120°, while the rank correlation decreased with the angle standard of 135° (Supplementary Table S1).Then, we considered the distance as the standard and then calculated the number of frames in which at least one of C7 or C8 is within the given distance [denoted as p (C7 or C8)], and only C8 is within the given distance [denoted as p (C8 only)] of all the track frames that meet the distance standard under different angle indicators (Supplementary Figures S9–S12). When the angle standard is 90°, 105°, 120°, and 135°, there is a good agreement between p (C8 only)/p (C7 or C8) and its product selectivity in the range of 3.6–4.0 (Supplementary Table S1), which is consistent with the optimal reaction distance obtained by the distance analysis. The results of the NAC angle analysis indicate that the tolerance of this kind of reaction to the angle is relatively broad, so the influence of distance on the occurrence of reaction is more important. Therefore, considering both distance and angle is not a significant improvement compared with only considering distance in this research, so the NAC angle is not a key factor that affects reaction selectivity.

**FIGURE 2:**
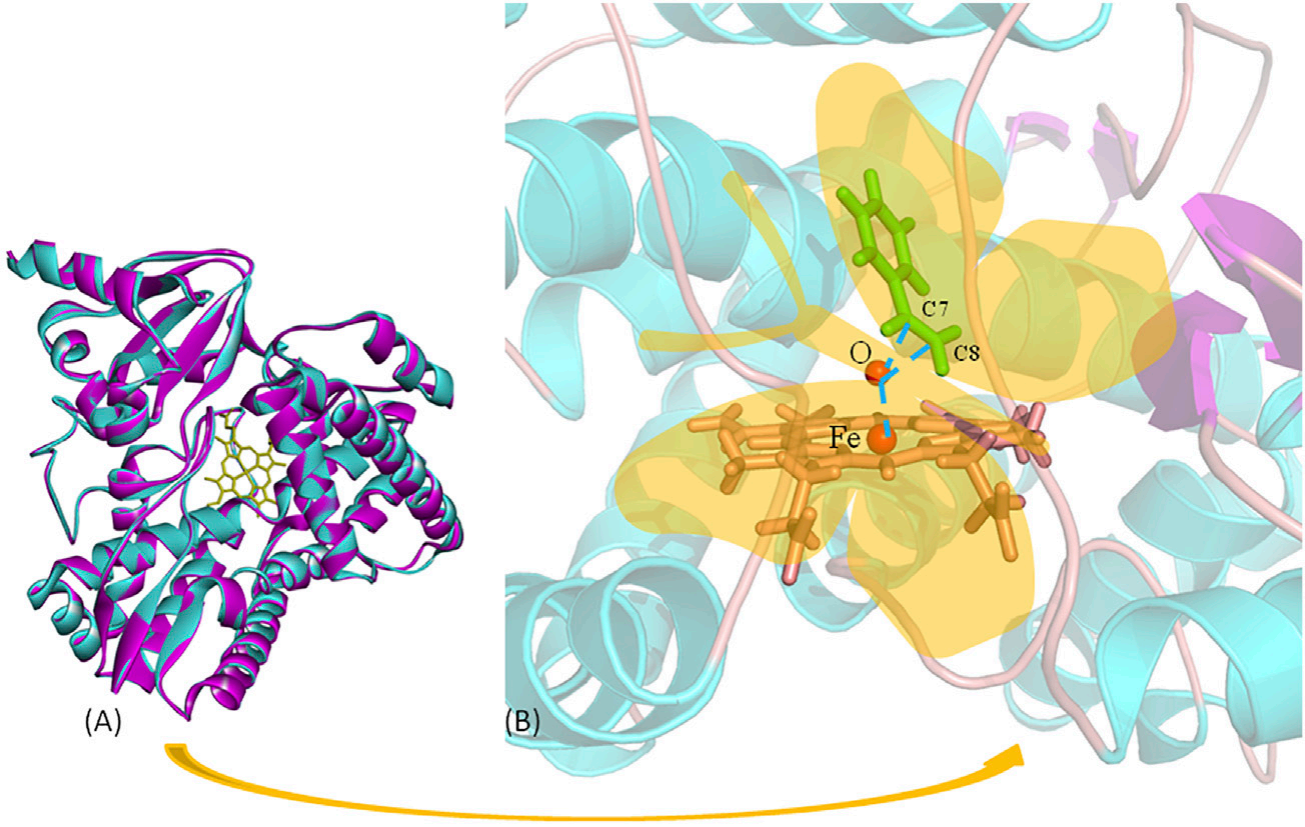
Butterfly model-based structure and computational analysis. **(A)** Overlay structure for aMOx (cyan) and the model (purple) CYP116B5 (PDB No.: 6RO8). **(B)** “Butterfly” catalytic model for P450 aMOx.

These data mentioned previously indicate that the following two key factors determine the AM selectivity of aMOx: the p (C8 only)/p (C7 or C8) and the distance from the reaction site C8 to the oxygen atom of the reaction active center Cpd I (C8-O). The ratio of p (C8 only)/p (C7 or C8) also showed a consistent upward trend when the AM selectivity increased. The distance from the reaction site C8 to the oxygen atom of the reaction active center Cpd I was maintained within 3–5 Å when the reaction occurred. Consequently, we proposed a butterfly catalysis model built on the aMOx reaction active site because the structure of the substrate and Cpd I was like a butterfly (Figure 2B). We hope that our model will provide a visual understanding of our rational design mechanism for experimental scientists.

### Rational Design for Anti-Markovnikov Selectivity of aMOx Based on the “Butterfly” Catalytic Model

**FIGURE 3:**
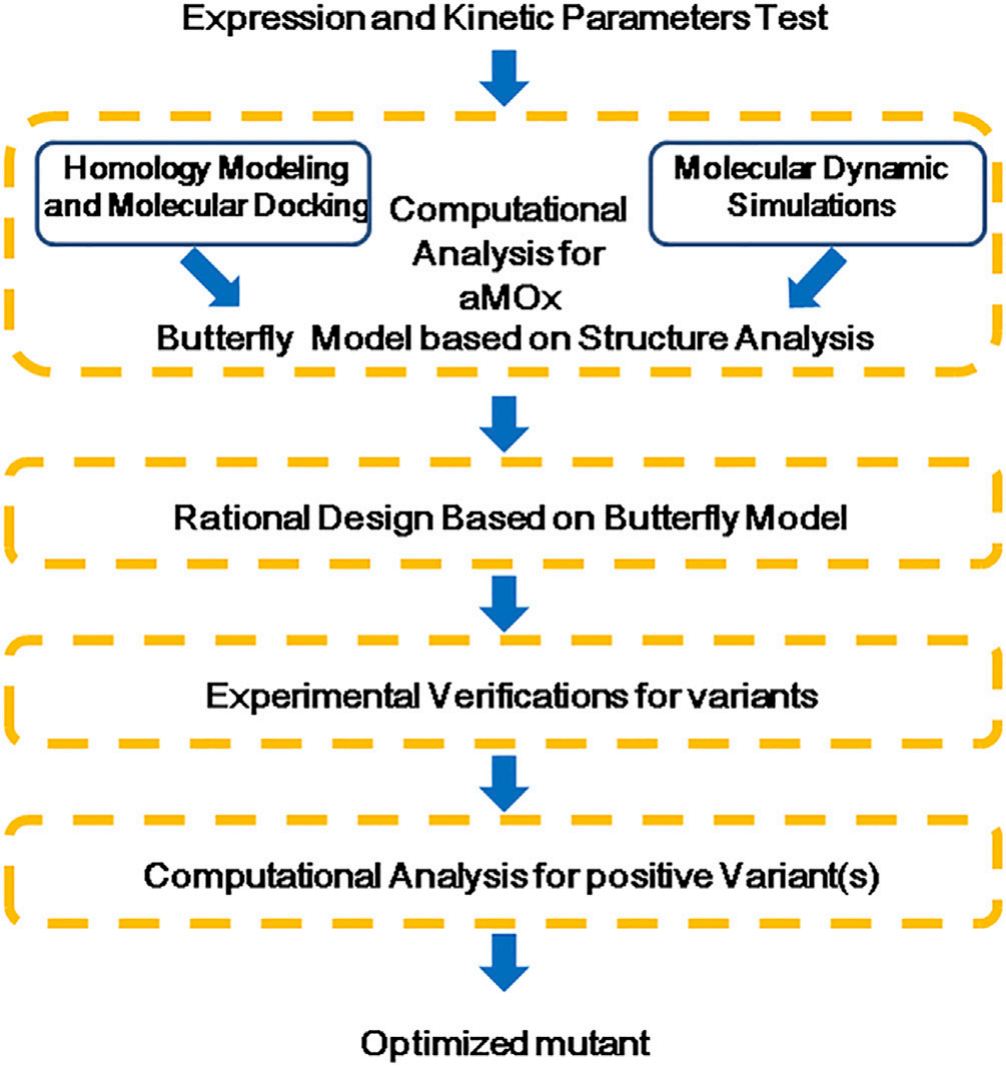
Flow chart of a rational design for aMOx.

The whole procedure of the aMOx rational design is shown in Figure 3. According to the results of the preliminary computational analysis, C8-O and p (C8 only)/p (C7 or C8) are important factors. Then, we investigated the binding energy contribution of each residue in aMOx to the substrate molecule when the substrate was close to the reaction center by introducing a distance constraint so that C8 in the substrate is at a distance of 3 Å from the oxygen atom in Cpd I during the simulation, and the results are shown in Table 1 by the MMGBSA binding energy decomposition method.

**TABLE 1:** MMGBSA binding energy decomposition of the CYP450 wild type binding with styrene (all units are in kcal/mol).

Residue	MMGBSA binding energy				
	VDW	ELE	GB	NP	Tot
279Ala	−0.75 ± 0.27	−0.09 ± 0.04	0.08 ± 0.04	−0.11 ± 0.02	−0.86 ± 0.29
123Ile	−0.67 ± 0.27	0.00 ± 0.02	0.00 ± 0.01	−0.11 ± 0.02	−0.77 ± 0.30
329Trp	−0.82 ± 0.10	−0.05 ± 0.01	0.23 ± 0.02	−0.08 ± 0.02	−0.72 ± 0.08
97Leu	−0.52 ± 0.09	0.00 ± 0.01	0.04 ± 0.01	−0.03 ± 0.01	−0.52 ± 0.10
278Val	−0.39 ± 0.31	−0.04 ± 0.03	−0.01 ± 0.03	−0.02 ± 0.02	−0.46 ± 0.37
275Ala	−0.51 ± 0.05	0.07 ± 0.05	0.04 ± 0.02	−0.04 ± 0.01	−0.44 ± 0.04
429Phe	−0.45 ± 0.16	−0.02 ± 0.03	0.10 ± 0.05	−0.05 ± 0.02	−0.42 ± 0.15
211Trp	−0.51 ± 0.22	−0.02 ± 0.02	0.16 ± 0.06	−0.04 ± 0.02	−0.42 ± 0.18
276Ile	−0.39 ± 0.23	0.02 ± 0.01	0.00 ± 0.01	−0.03 ± 0.04	−0.41 ± 0.27
283Thr	−0.35 ± 0.15	−0.04 ± 0.03	0.09 ± 0.07	−0.05 ± 0.02	−0.35 ± 0.15
All	−7.26 ± 0.76	−0.25 ± 0.08	1.20 ± 0.12	−0.63 ± 0.04	−6.94 ± 0.70

The total binding energy of the protein (except its Cpd I group) to the styrene is listed as “All” row in the table, and the top 10 protein residues that contribute most to the substrate’s binding are also listed in the table based on their energy contributions from large (more negative) to small (less negative). The “VDW,” “ELE,” “GB,” and “NP” columns are the van der Waals, electrostatic, generalized Born solvation, and nonpolar solvation energy, respectively, and the “Tot” column is the sum of these four energy terms. As can be seen, since styrene is nonpolar, the VDW energy plays a major role in binding, while the contributions of ELE, GB, and NP terms are all limited.

**FIGURE 4:**
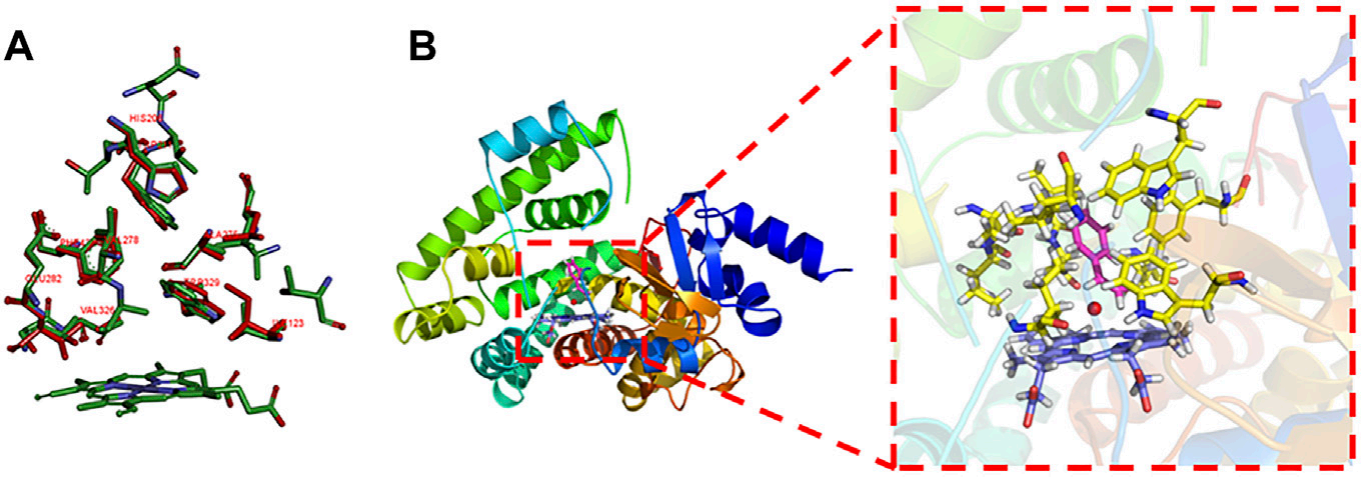
**(A)** Analysis of the aMOx substrate’s binding pocket. Residuals consisting of the aMOx substrate-binding channel are shown in red and those of CYP116B5 are shown in green. **(B)** Key residuals that affect the AM selectivity of aMOx. Key residuals are marked in yellow.

The key residuals that affect the AM selectivity of the aMOx are shown in Figure 4B. The resolution of the substrate binding channel of the template CYP116B5 used in the homology modeling part has been reported by previous studies. We performed a structural comparison between the binding pocket of aMOx and CYP116B5. The results indicated that the substrate-binding channel of aMOx is largely identical to that of CYP116B5, both being a four-layered substrate binding channel composed of non-polar hydrophobic amino acids (Figure 4A). In addition, Ile123, His206, Trp211, Ala275, Val278, Trp329, and Phe429 were all on the substrate-binding channel among the key sites affecting the substrate binding conformation of styrene analyzed by MMGBSA mentioned previously. Overall, it was confirmed that AM selectivity of P450 aMOx could be altered by altering its substrate binding channel.

### Experimental Validation of Anti-Markovnikov Selectivity of aMOx and Mutants

To verify the AM selectivity of aMOx and its variants, we performed an experimental alanine scan for the aforementioned substrate-binding key residues. Seamless cloning was achieved for aMOx A279G, I123A, W329A, L97A, V278A, A275G, F429A, W211A, I276A, and T283A. The primers used are listed in Table 2.

**TABLE 2:** Primers used for the construction of aMOx variants.

Mutant	Primer	Primer sequence
—	aMOx-F	ATA​CAT​ATG​GAG​CGC​ACT​GCA​AAT
	aMOx-R	ATA​AAG​CTT​CTC​GAG​CAG​TGC​CAG
L97A	L97A-F	CGT​AAC​GCT​GCG​GAA​AAA​ATC​ACT​CCG​CTG​ACC
	L97A-R	GAT​TTT​TTC​CGC​AGC​GTT​ACG​CGG​GCT​GAA​CAG
I123A	I123A-F	CAT​GCC​ATG​GCT​AAC​GAA​GAC​GAA​CCA​GTT
	I123A-R	GTC​TTC​GTT​AGC​CAT​GGC​ATG​GTT​CAG​TGC
W211A	W211A-F	GTC​AGC​ACC​GCG​GGT​AAA​CCG​ACC​GAT​GAG​CAG
	W211A-R	CGG​TTT​ACC​CGC​GGT​GCT​GAC​GCT​GTG​TGC​GAC
A275G	A275G-F	ATG​ATG​ATG​GGT​ATC​ATC​GTT​GCG​GCA​CAC​GAG
	A275G-R	AAC​GAT​GAT​ACC​CAT​CAT​CAT​GGA​GTG​AAC​ATA
I276A	I276A-F	ATG​ATG​GCG​GCG​ATC​GTT​GCG​GCA​CAC​GAG​ACC
	I276A-R	CGC​AAC​GAT​CGC​CGC​CAT​CAT​CAT​GGA​GTG​AAC
V278A	V278A-F	GCG​ATC​ATC​GCG​GCG​GCA​CAC​GAG​ACC​ACC​AGC
	V278A-R	GTG​TGC​CGC​CGC​GAT​GAT​CGC​CAT​CAT​CAT​GGA
A279G	A279G-F	ATC​ATC​GTT​GGT​GCA​CAC​GAG​ACC​ACC​AGC​CTG
	A279G-R	CTC​GTG​TGC​ACC​AAC​GAT​GAT​CGC​CAT​CAT​CAT
T283A	T283A-F	GCA​CAC​GAG​GCG​ACC​AGC​CTG​GCC​TCT​GCA​GGT
	T283A-R	CAG​GCT​GGT​CGC​CTC​GTG​TGC​CGC​AAC​GAT​GAT
W329A	W329A-F	GTT​ATG​GCA​GCG​CGT​CGT​CAA​GCT​ACG​GCT​GCC
	W329A-R	TTG​ACG​ACG​CGC​TGC​CAT​AAC​GGA​GCC​GCT​ATA
F429A	F429A-F	AAC​ACC​AGC​GCG​CGT​GGT​CCG​GAT​CAT​GTG​TGG
	F429A-R	CGG​ACC​ACG​CGC​GCT​GGT​GTT​GGA​CAG​GTA​AGT

**FIGURE 5:**
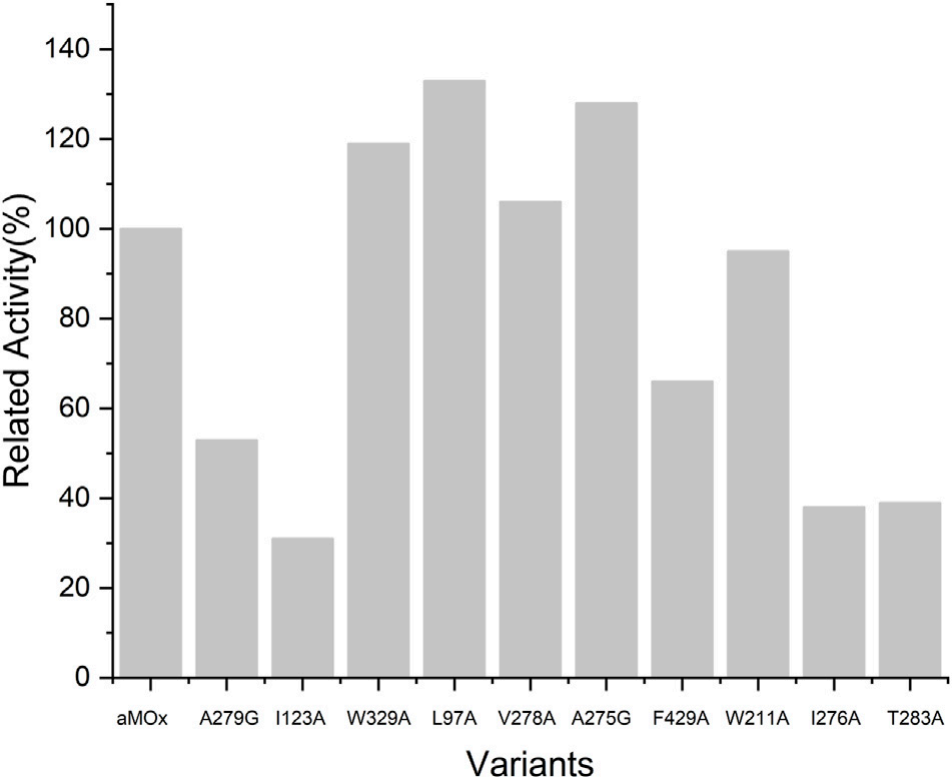
Relative activity of variants and aMOx. All samples were performed at least twice.

As all of the aforementioned mutations were soluble expressions, we examined the specific activity of these mutants before the assay for mutant anti-Markovnikov selectivity (Figure 5). All 10 mutations had a specific activity. Compared to the wild type, V278A, L97A, A275G, and W329A all had elevated specific activities of 6%, 33%, 19%, and 29%, respectively. We performed quantitative assays of the AM selectivity products for all mutants. The AM selectivity of the aforementioned mutants was experimentally verified with styrene as the substrate and Purpald derivatization reaction, and the experimental results showed that the AM selectivity of A275G appeared to be significantly enhanced to >99% (Table 3).

**TABLE 3:** Specific activity and AM selectivity of aMOx and its variants. All samples were performed at least thrice.

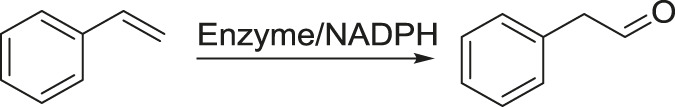		
Mutant	U/mg	AM selectivity (%)
aMOx	10.45	81 (reported)
A279G	6.37	76
I123A	4.13	41
W329A	11.98	68
L97A	13.66	29
V278A	13.99	59
A275G	13.17	>99
F429A	8.66	47
W211A	12.56	45
I276A	5.05	37
T283A	5.14	38

### Computational Analysis of aMOx A275G

**FIGURE 6:**
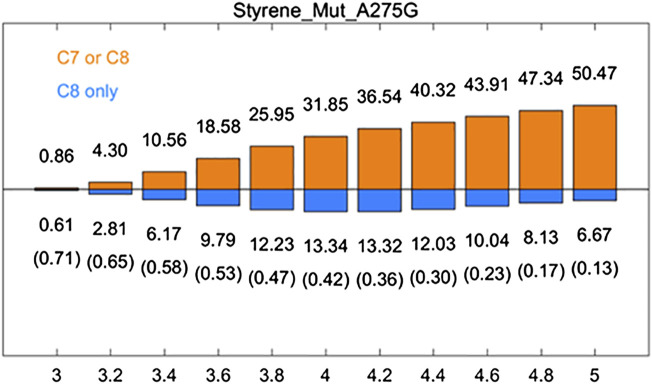
Rate of p (C8 only)/p (C7 or C8) of aMOx A275G. p (C8 only) are shown in the blue bar, and p (C7 or C8) are shown in the orange bar. The ratios of p (C8 only)/p (C7 or C8) are also listed in the brackets. All results are the averages of the corresponding ten 30 ns aMD trajectories.

A275G is located on the substrate-binding channel of aMOx (Figure 4). To analyze the increased AM selectivity of A275G, we performed molecular dynamic simulations for A275G (Figure 6). p (C8 only)/p (C7 or C8) values for A275G were 0.53, 0.47, and 0.42, whereas the distance thresholds were 3.6, 3.8, and 4.0 Å. When analyzed in conjunction with round 1, round 2, round 8, and aMOx in the previous design, these ratios have a strong correlation with the AM selectivity of the corresponding systems. As Table 4 shows, the Spearman ranking correlation coefficient between the two systems was 1 with a 3.8 Å distance threshold. The Spearman ranking correlation coefficient was 0.97. This revealed that the AM selectivity of round 1, round 2, round 8, aMOx, and A275G changed in the same trend, which also proved that p (C8 only)/p (C7 or C8) and the distance between C8 and heme oxygen are two key factors in the AM selective design of an aMOx product. Hence, this strategy provides guidance for the rational design of this class of enzymes.

**TABLE 4:** Inverse martensite selectivity of the system and the ratio of p (C8 only)/p (C7 or C8) in the simulated trajectories of aMD at different distance thresholds.

Enzyme	Anti-Markovnikov	Ratio p (C8 only)/p (C7 or C8)		
	Selectivity	Maxdist = 3.6	Maxdist = 3.8	Maxdist = 4.0
Round 1	0.45	0.34	0.29	0.25
Round 2	0.55	0.34	0.30	0.26
Round 8	0.76	0.35	0.33	0.30
aMOx	0.81	0.42	0.36	0.30
A275G	0.99	0.53	0.47	0.42
Spearman ranking power		0.97	1.00	0.97

## Conclusion

The AM oxidation of aromatic olefins into corresponding aromatic aldehydes is an important synthetic route in the industry. Using computational methods combined with experiments, we revealed two key factors that influence the substrate’s AM selectivity of P450 aMOx as follows: 1) distance and 2) frequency of substrate reaction sites within a reasonable distance from the reaction. Based on these key factors, we have proposed a “butterfly” catalytic model. Finally, we successfully designed the mutant aMOx A275G with an AM selectivity of more than 99%. The results of this study are expected to contribute to the rational design of AM selectivity for enzyme-catalyzed AM oxidation reactions.

## Data Availability

The original contributions presented in the study are included in the article/Supplementary Material, further inquiries can be directed to the corresponding authors.
