# Supplementary material for: Rational Design of P450 aMOx for Improving Anti-Markovnikov Selectivity Based on the “Butterfly” Model
Source: Front Mol Biosci. 2022 May 23;9:888721. doi: 10.3389/fmolb.2022.888721 (PMC9168652; doi:10.3389/fmolb.2022.888721)
Supplement: Supplementary file 1 [file DataSheet1.docx]

Supplementary Material

# Supplementary Figures


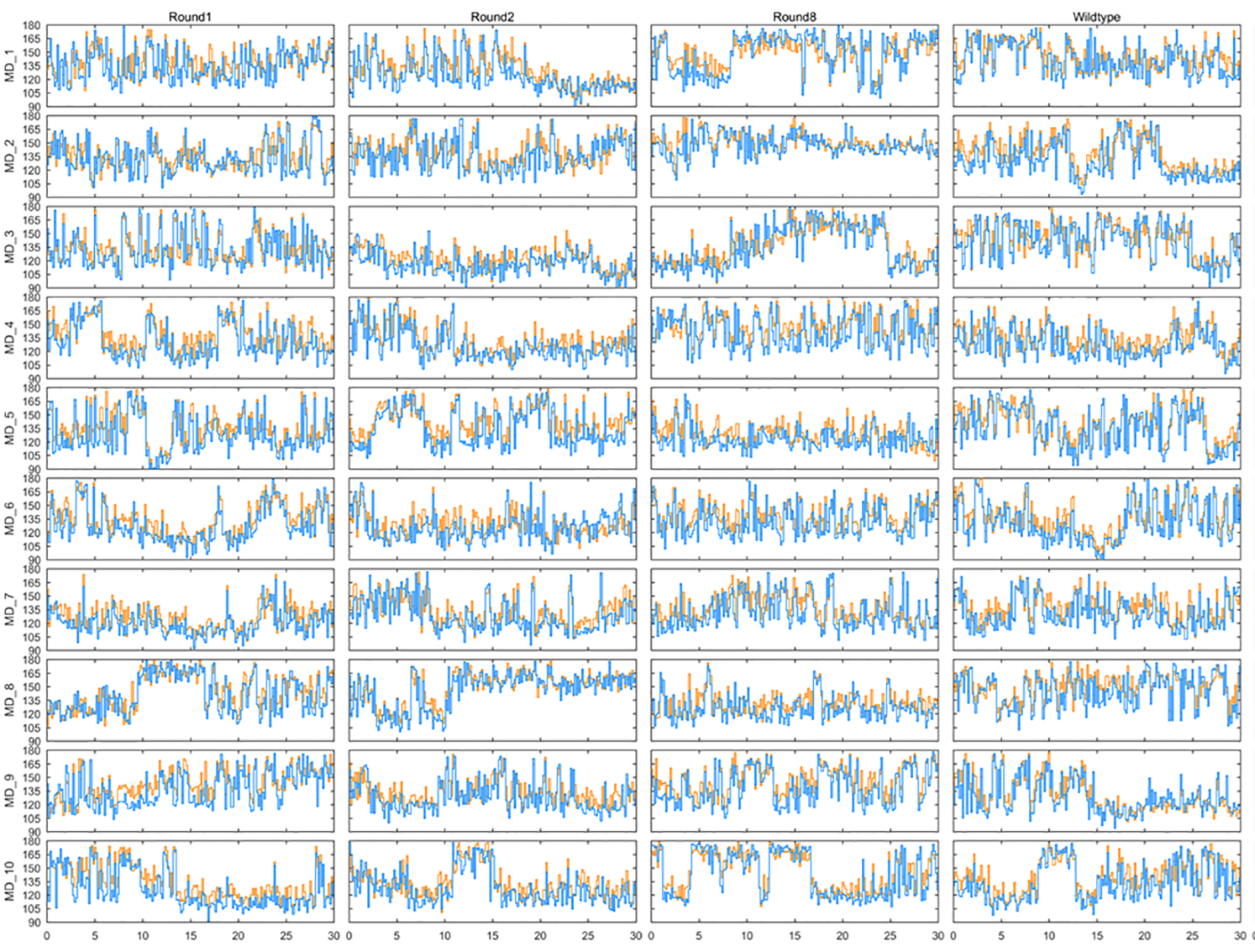


**Supplementary Figure 1.** Angles (y-axis, in degree) formed by the C8 (blue) or the C7 (Orange) of styrene and the oxygen and iron atoms of Cpd I in each trajectory of each enzyme as a function of the simulation time (x-axis, in ns)


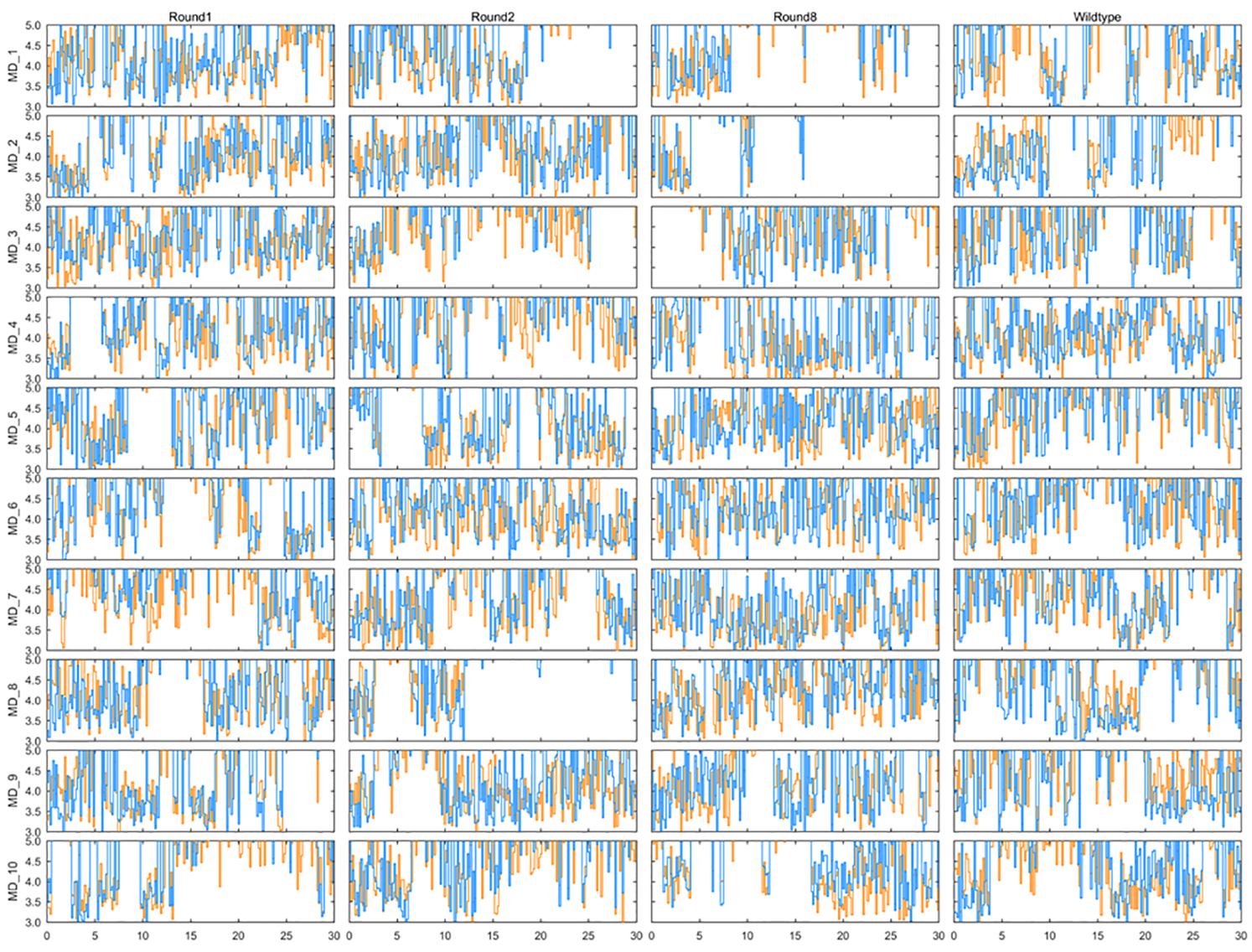


**Supplementary Figure 2.** Distances (y-axis, in Å) between C8 (blue) or C7 (Orange) of styrene to the oxygen of Cpd I in each trajectory of each enzyme as a function of the simulation time (x-axis, in ns).


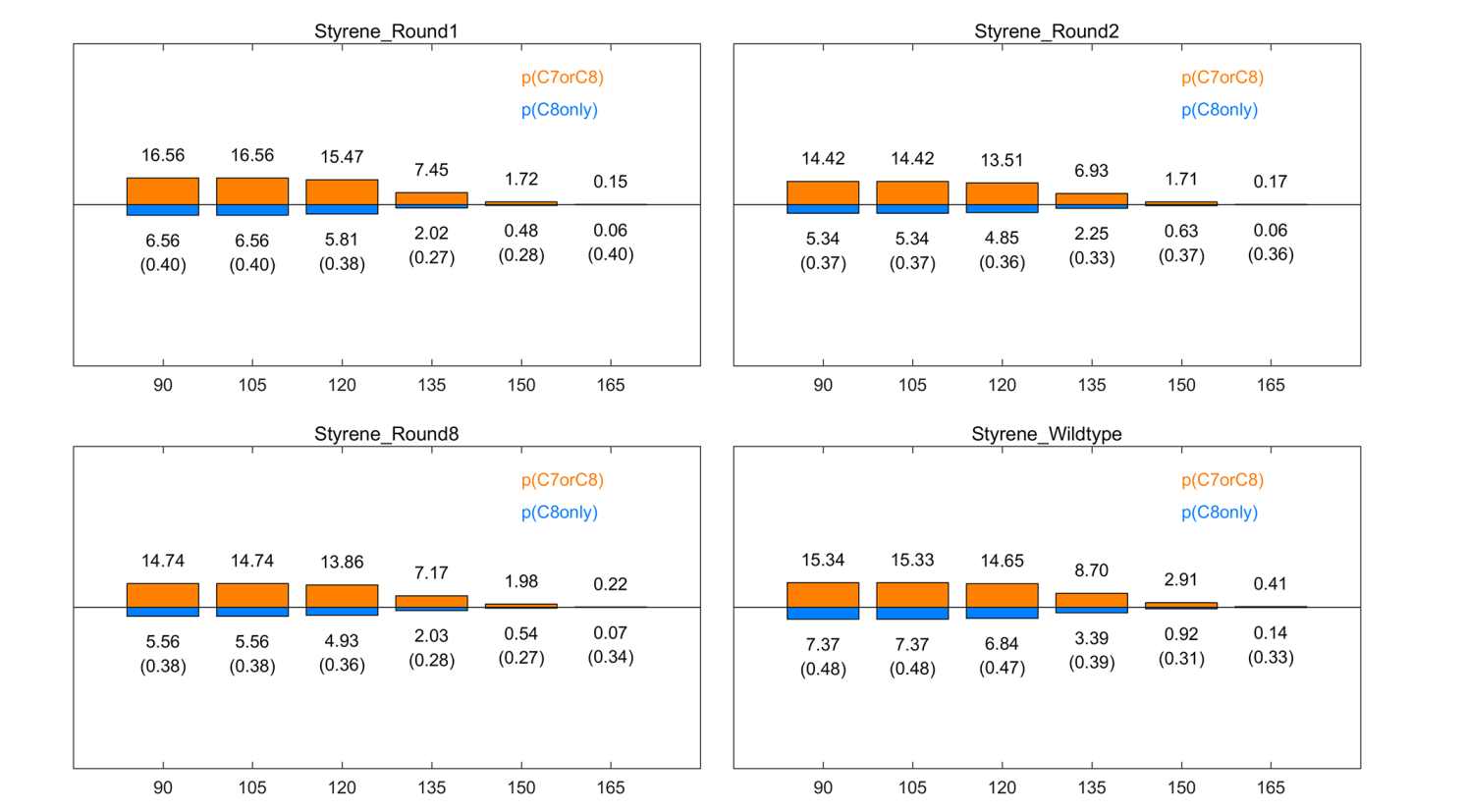


**Supplementary Figure 3.** Angle frequency statistics of C7/C8-O-Fe when the distance between C7/C8 and heme oxygen is 3.4Å. The percentages (p(C7orC8), orange) of the frames that have at least one angle are more than given degree (x-axis, in °) among all the aMD frames, and the percentages (p(C8only), blue) of the frames that only C8 is within 3.4Å. The ratios of p(C8only)/p(C7orC8) are also listed in the brackets. All results are the averages of the corresponding ten 30 ns aMD trajectories.


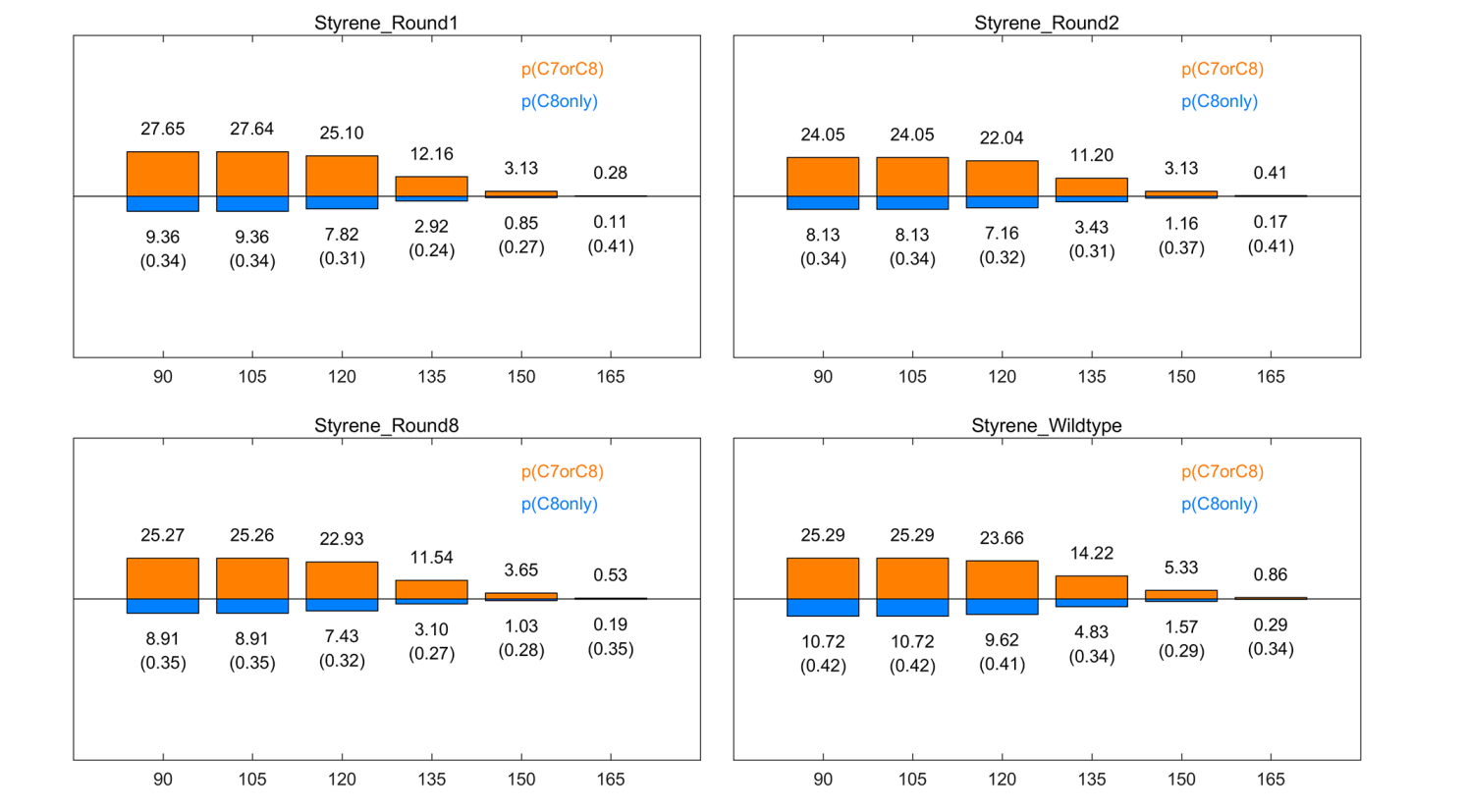


**Supplementary Figure 4.** Angle frequency statistics of C7/C8-O-Fe when the distance between C7/C8 and heme oxygen is 3.6Å. The percentages (p(C7orC8), orange) of the frames that have at least one angle are more than given degree (x-axis, in °) among all the aMD frames, and the percentages (p(C8only), blue) of the frames that only C8 is within 3.6Å. The ratios of p(C8only)/p(C7orC8) are also listed in the brackets. All results are the averages of the corresponding ten 30 ns aMD trajectories.


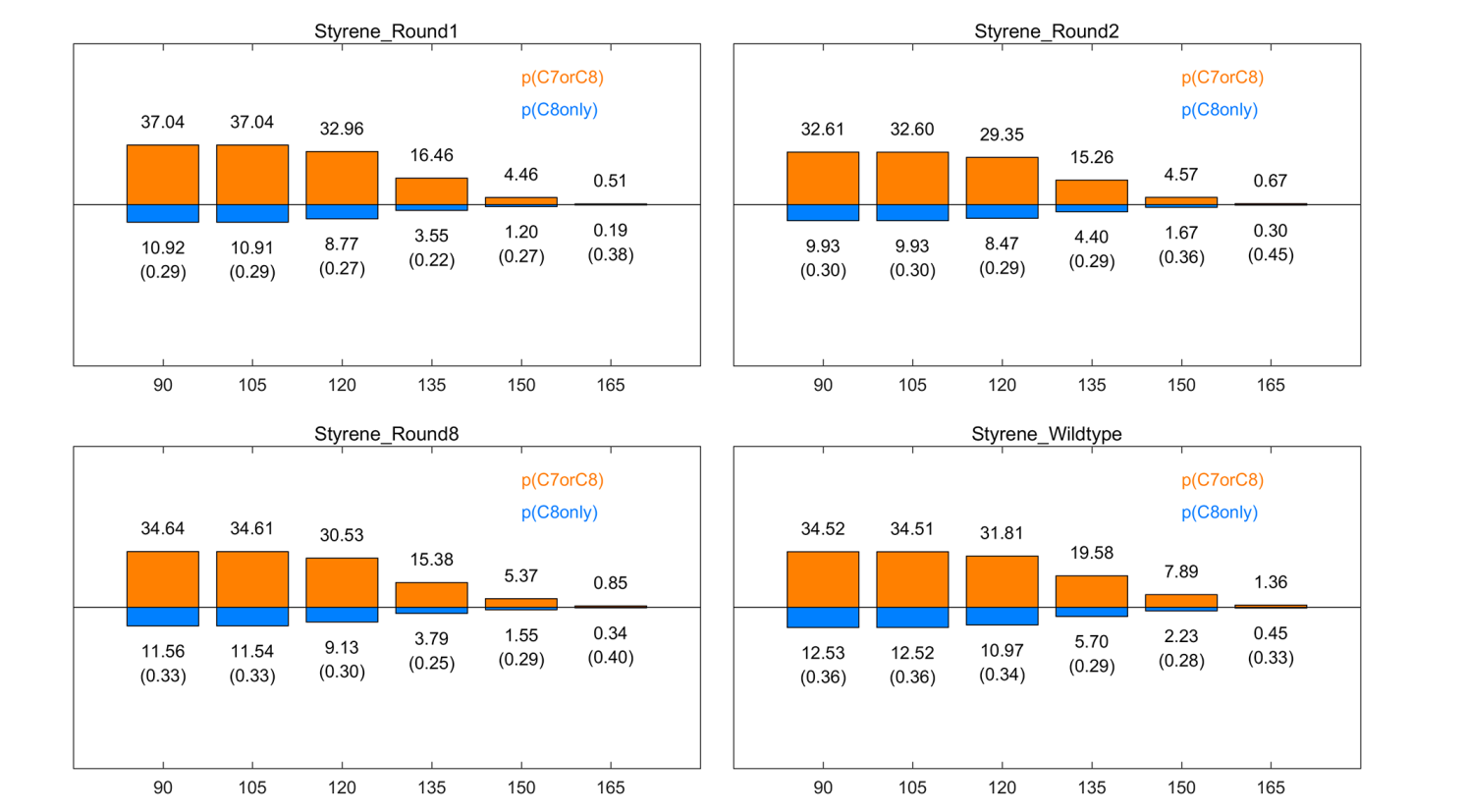


**Supplementary Figure 5.** Angle frequency statistics of C7/C8-O-Fe when the distance between C7/C8 and heme oxygen is 3.8Å. The percentages (p(C7orC8), orange) of the frames that have at least one angle are more than given degree (x-axis, in °) among all the aMD frames, and the percentages (p(C8only), blue) of the frames that only C8 is within 3.8Å. The ratios of p(C8only)/p(C7orC8) are also listed in the brackets. All results are the averages of the corresponding ten 30 ns aMD trajectories.


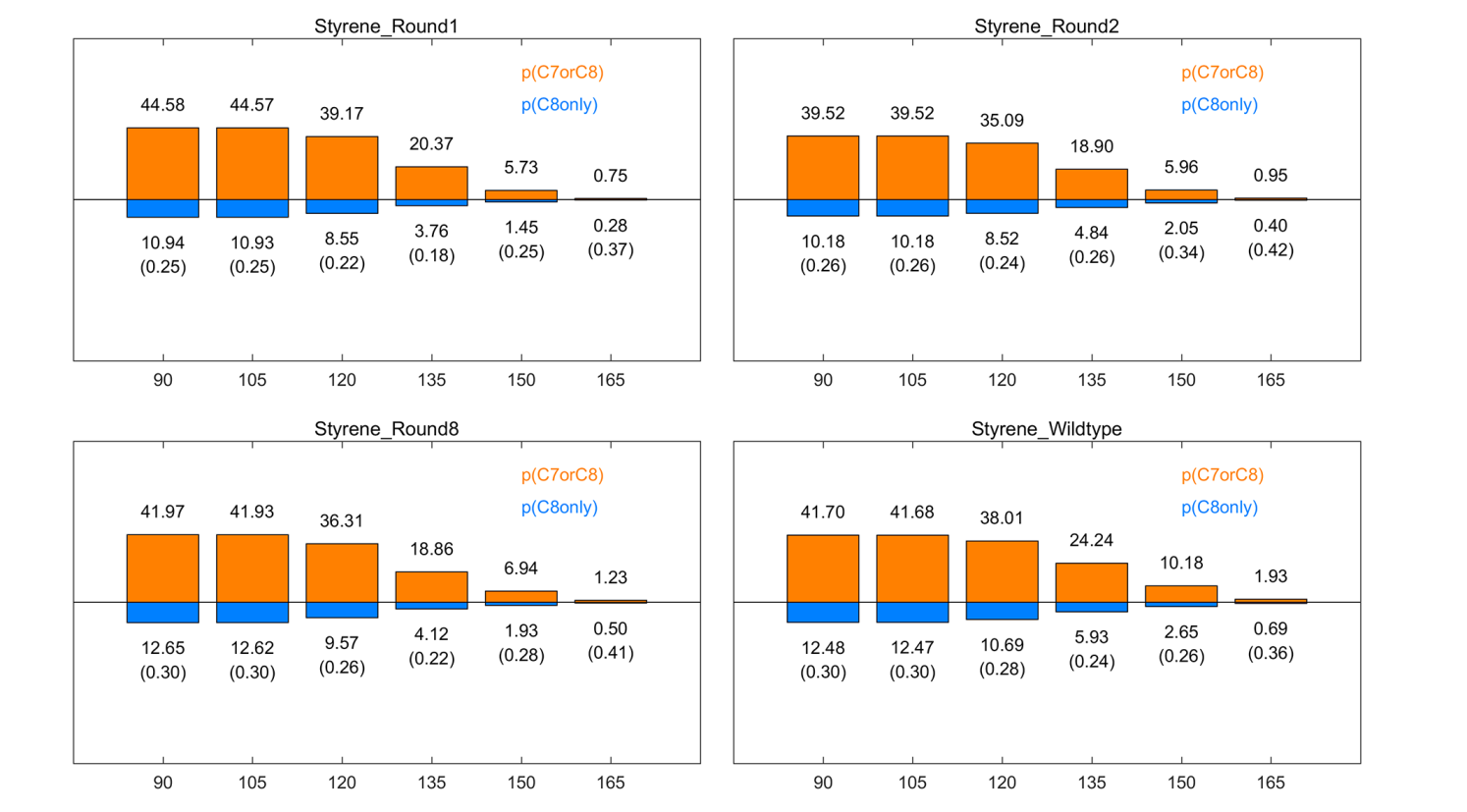


**Supplementary Figure 6.** Angle frequency statistics of C7/C8-O-Fe when the distance between C7/C8 and heme oxygen is 4.0Å. The percentages (p(C7orC8), orange) of the frames that have at least one angle are more than given degree (x-axis, in °) among all the aMD frames, and the percentages (p(C8only), blue) of the frames that only C8 is within 4.0Å. The ratios of p(C8only)/p(C7orC8) are also listed in the brackets. All results are the averages of the corresponding ten 30 ns aMD trajectories.


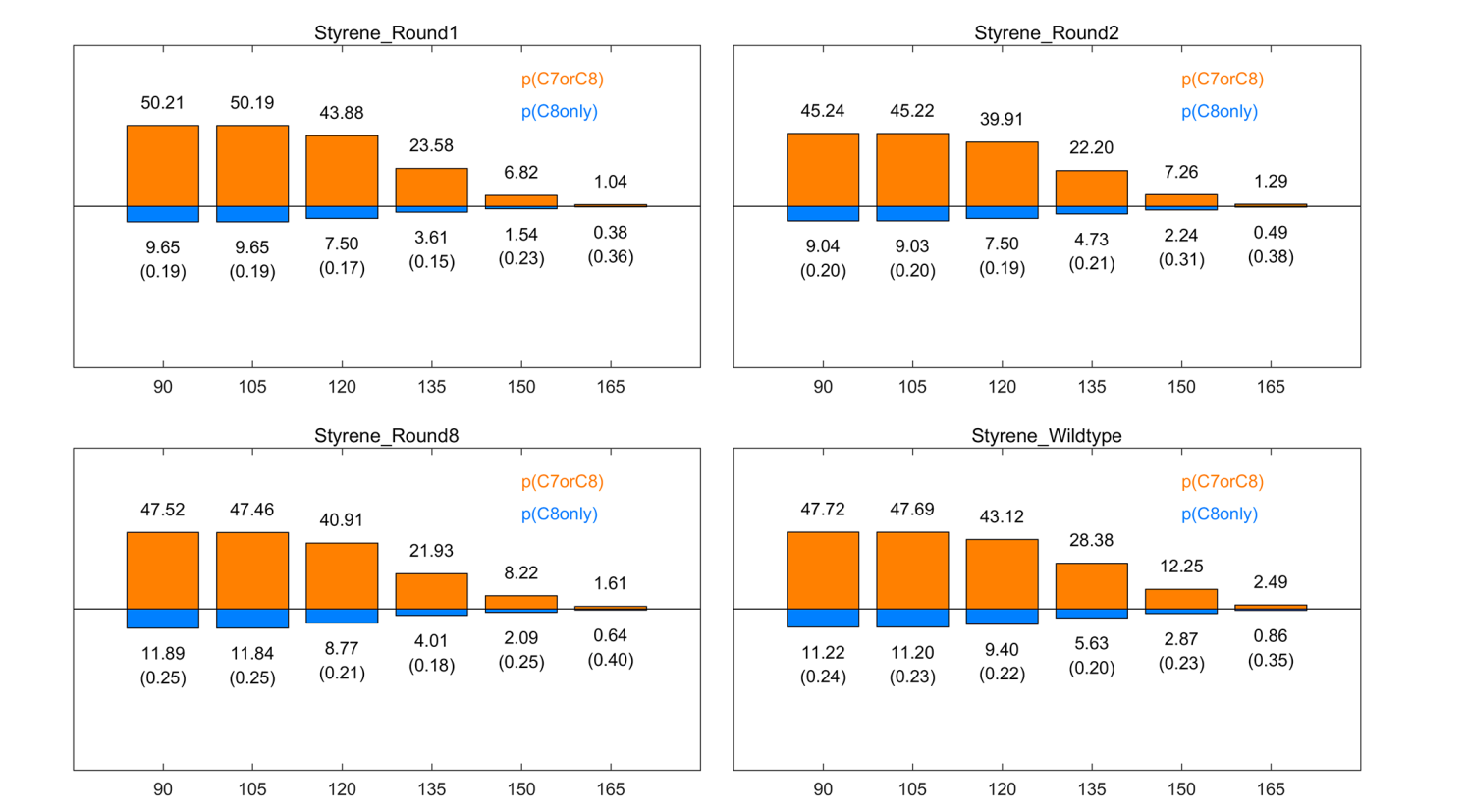


**Supplementary Figure 7.** Angle frequency statistics of C7/C8-O-Fe when the distance between C7/C8 and heme oxygen is 4.2Å. The percentages (p(C7orC8), orange) of the frames that have at least one angle are more than given degree (x-axis, in °) among all the aMD frames, and the percentages (p(C8only), blue) of the frames that only C8 is within 4.2Å. The ratios of p(C8only)/p(C7orC8) are also listed in the brackets. All results are the averages of the corresponding ten 30 ns aMD trajectories.


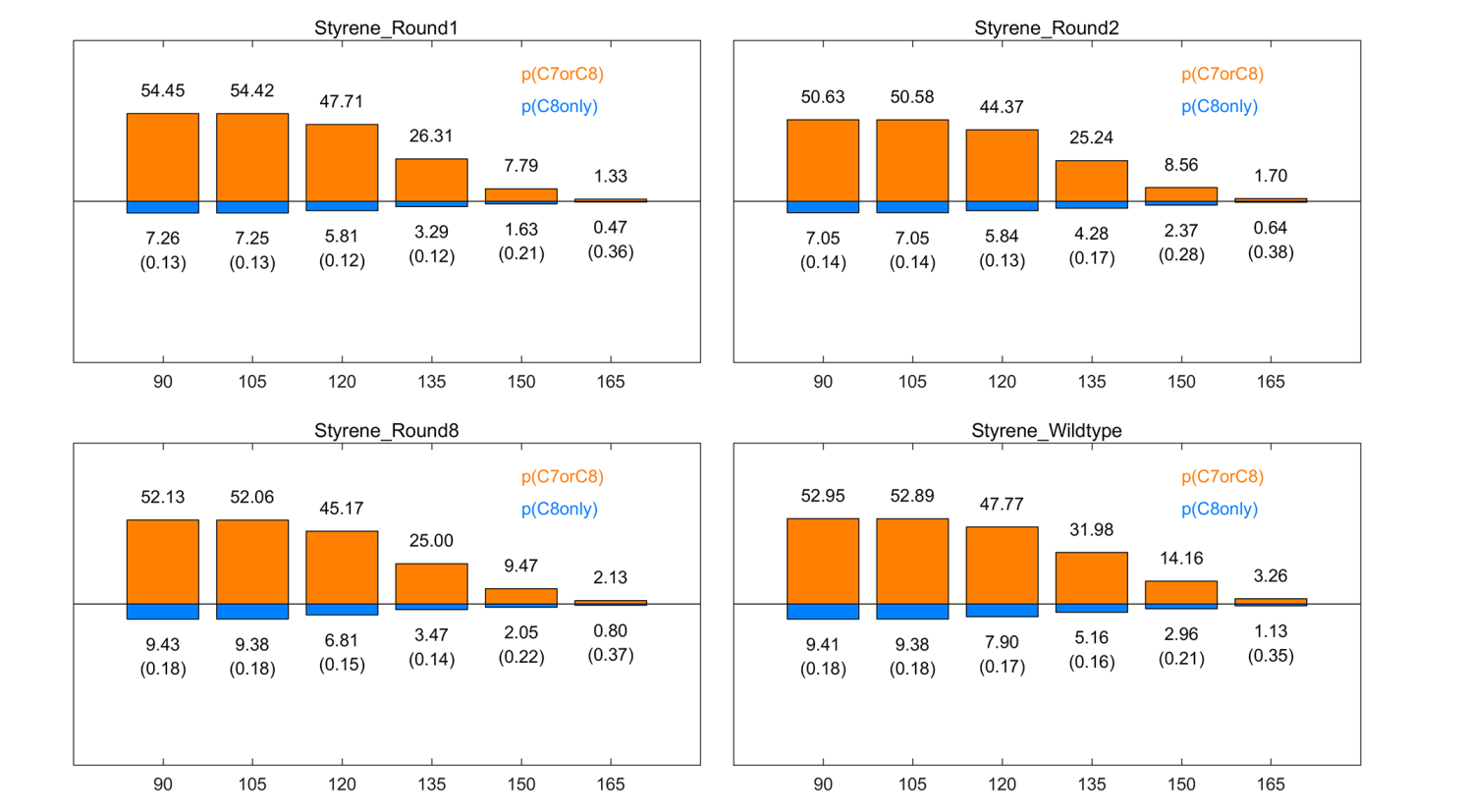


**Supplementary Figure 8.** Angle frequency statistics of C7/C8-O-Fe when the distance between C7/C8 and heme oxygen is 4.4Å. The percentages (p(C7orC8), orange) of the frames that have at least one angle are more than given degree (x-axis, in °) among all the aMD frames, and the percentages (p(C8only), blue) of the frames that only C8 is within 4.4Å. The ratios of p(C8only)/p(C7orC8) are also listed in the brackets. All results are the averages of the corresponding ten 30 ns aMD trajectories.


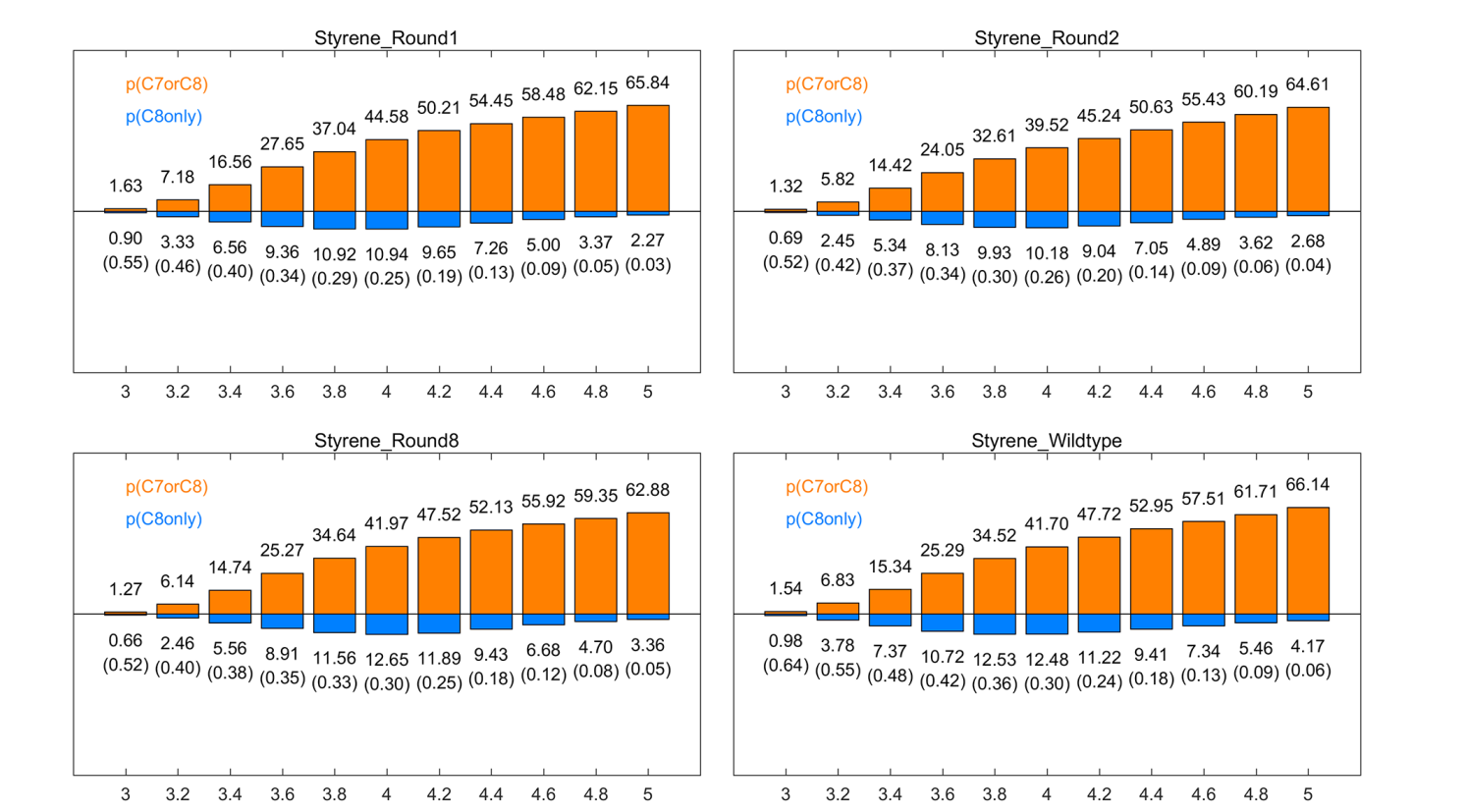


**Supplementary Figure 9.** Distance frequency statistics of C7/C8-O when C8-O-Fe is 90°. The percentages (p(C7orC8), orange) of the frames that have at least one carbon (of the styrene’s Position1 and Position2 carbons) within given distance (x-axis, in Å) to the Cpd I oxygen among all the aMD frames, and the percentages (p(C8only), blue) of the frames that only the Position1 carbon of styrene vinyl are within the given distance. The ratios of p(C8only)/p(C7orC8) are also listed in the brackets. All results are the averages of the corresponding ten 30 ns aMD trajectories.


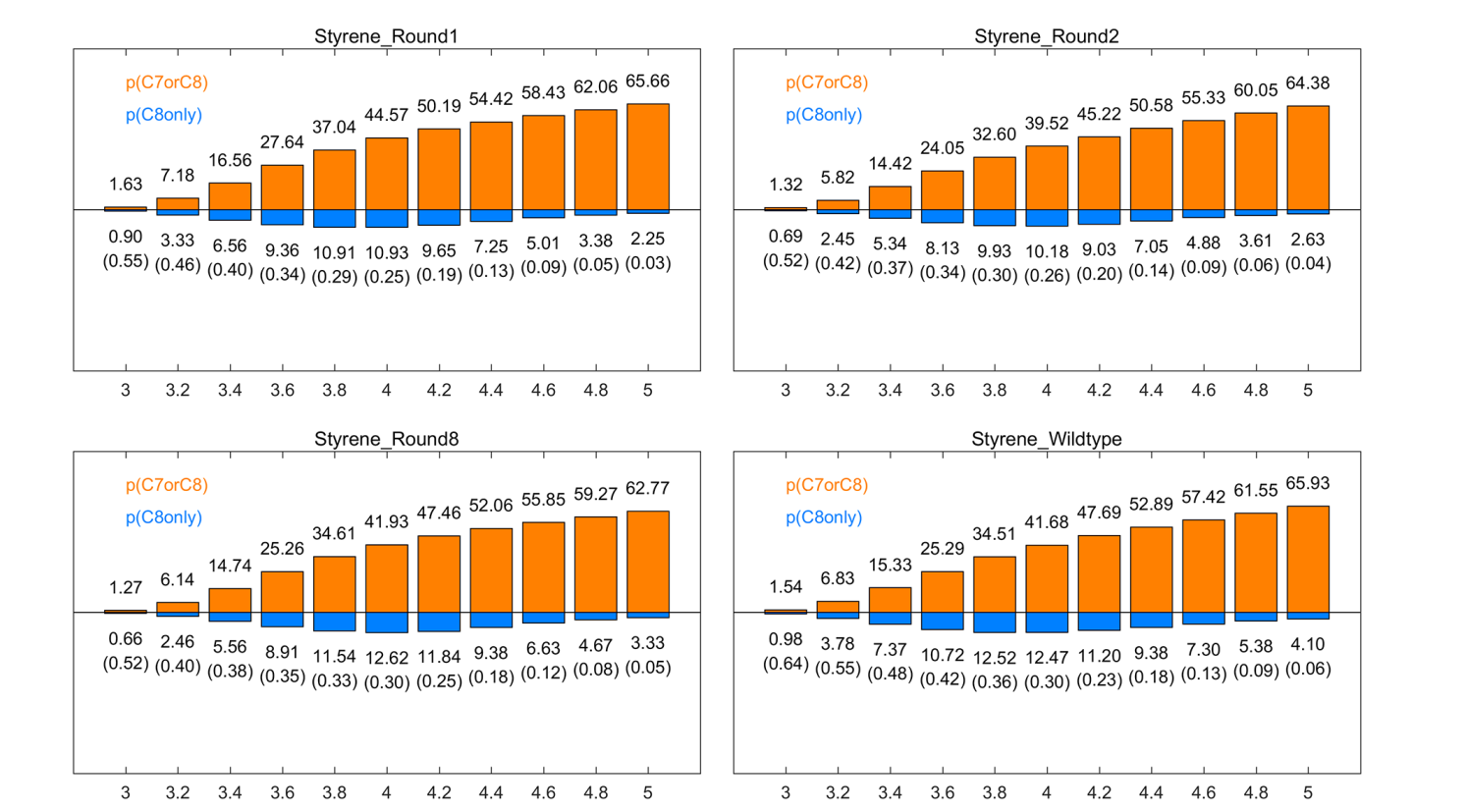


**Supplementary Figure 10.** Distance frequency statistics of C7/C8-O when C8-O-Fe is 105°. The percentages (p(C7orC8), orange) of the frames that have at least one carbon (of the styrene’s Position1 and Position2 carbons) within given distance (x-axis, in Å) to the Cpd I oxygen among all the aMD frames, and the percentages (p(C8only), blue) of the frames that only the Position1 carbon of styrene vinyl are within the given distance. The ratios of p(C8only)/p(C7orC8) are also listed in the brackets. All results are the averages of the corresponding ten 30 ns aMD trajectories.


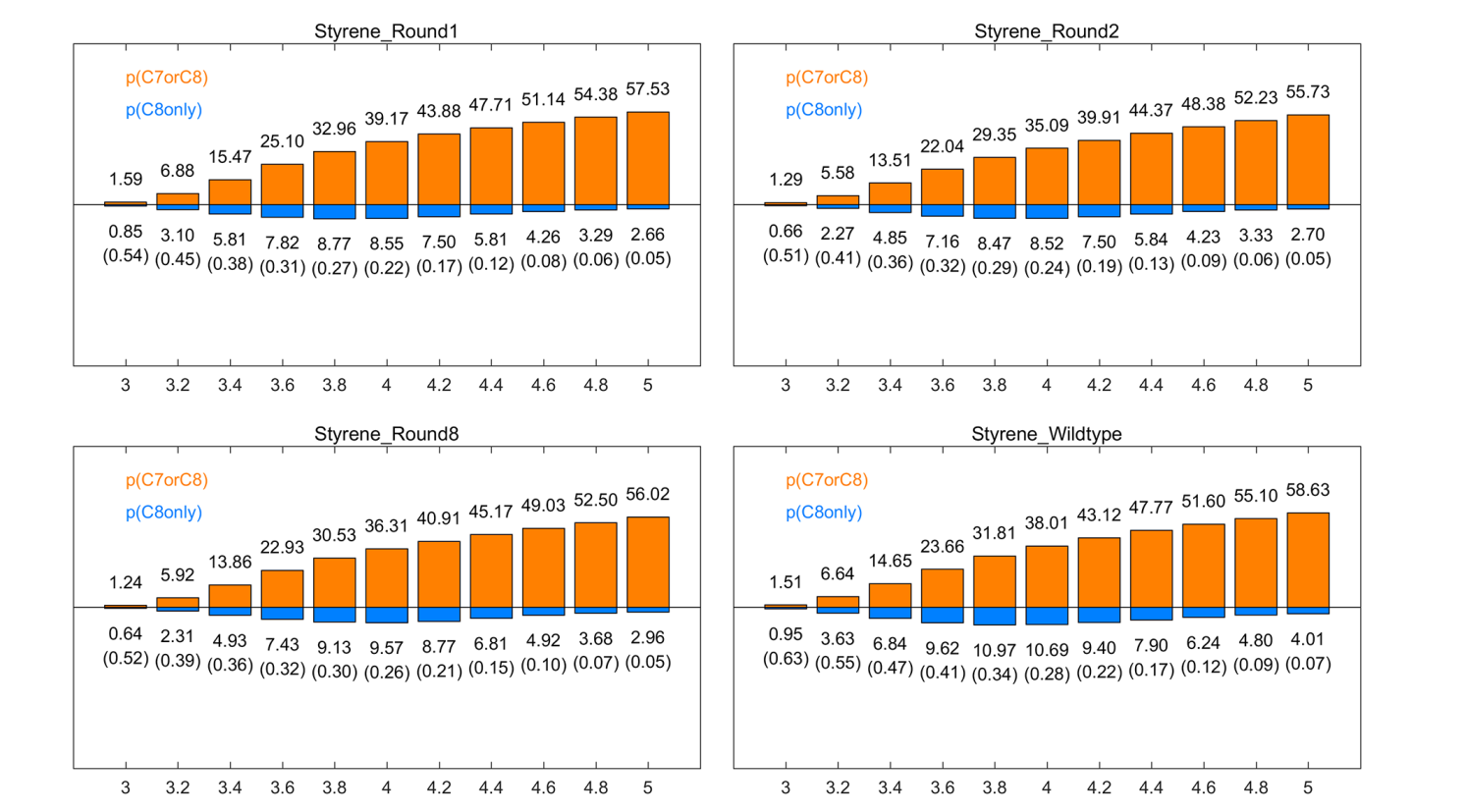


**Supplementary Figure 11.** Distance frequency statistics of C7/C8-O when C8-O-Fe is 120°. The percentages (p(C7orC8), orange) of the frames that have at least one carbon (of the styrene’s Position1 and Position2 carbons) within given distance (x-axis, in Å) to the Cpd I oxygen among all the aMD frames, and the percentages (p(C8only), blue) of the frames that only the Position1 carbon of styrene vinyl are within the given distance. The ratios of p(C8only)/p(C7orC8) are also listed in the brackets. All results are the averages of the corresponding ten 30 ns aMD trajectories.


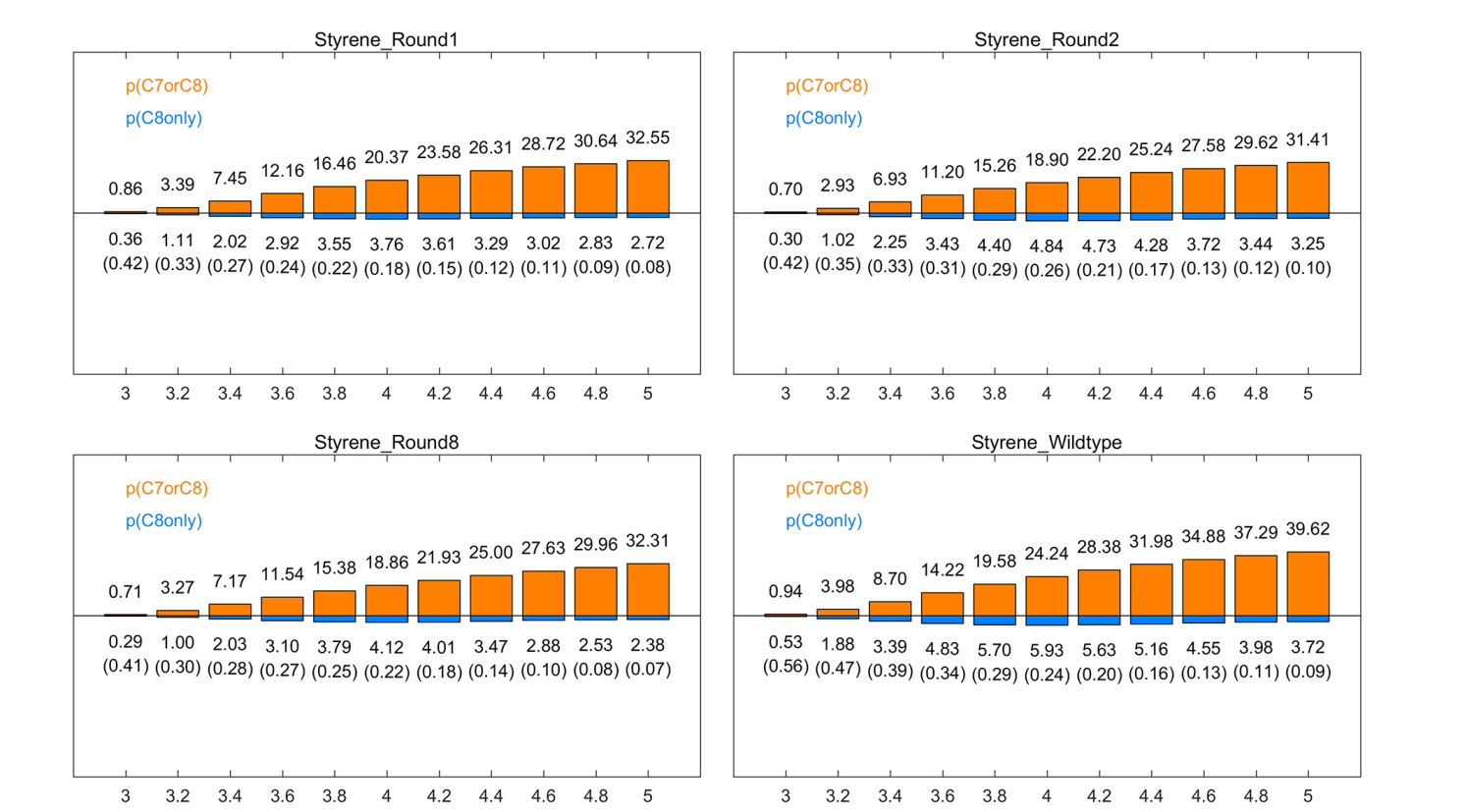


**Supplementary Figure 12.** Distance frequency statistics of C7/C8-O when C8-O-Fe is 135°. The percentages (p(C7orC8), orange) of the frames that have at least one carbon (of the styrene’s Position1 and Position2 carbons) within given distance (x-axis, in Å) to the Cpd I oxygen among all the aMD frames, and the percentages (p(C8only), blue) of the frames that only the Position1 carbon of styrene vinyl are within the given distance. The ratios of p(C8only)/p(C7orC8) are also listed in the brackets. All results are the averages of the corresponding ten 30 ns aMD trajectories.

# Supplementary Table

| **ID** | **p(C8only)/p(C7orC8)** | | **Round1** | **Round2** | **Round8** | **aMOx** | **A275G** | **Rank Spearman** |
| --- | --- | --- | --- | --- | --- | --- | --- | --- |
|  | **AM selectivity (%)** | | **0.45** | **0.55** | **0.76** | **0.81** | **0.99** | **ALL** |
| 1 | Angle_90 | Dist_3.0 | 55.1% | 51.9% | 52.1% | 63.8% | 71.1% | 0.70 |
| 2 | Angle_90 | Dist_3.2 | 46.3% | 42.1% | 40.1% | 55.4% | 65.4% | 0.60 |
| 3 | Angle_90 | Dist_3.4 | 39.6% | 37.1% | 37.7% | 48.1% | 58.4% | 0.70 |
| 4 | Angle_90 | Dist_3.6 | 33.9% | 33.8% | 35.3% | 42.4% | 52.7% | 0.90 |
| 5 | Angle_90 | Dist_3.8 | 29.5% | 30.5% | 33.4% | 36.3% | 47.1% | 1.00 |
| 6 | Angle_90 | Dist_4.0 | 24.5% | 25.8% | 30.1% | 29.9% | 41.9% | 0.90 |
| 7 | Angle_90 | Dist_4.2 | 19.2% | 20.0% | 25.0% | 23.5% | 36.5% | 0.90 |
| 8 | Angle_90 | Dist_4.4 | 13.3% | 13.9% | 18.1% | 17.8% | 29.8% | 0.90 |
| 9 | Angle_105 | Dist_3.0 | 55.1% | 51.9% | 52.1% | 63.8% | 71.1% | 0.70 |
| 10 | Angle_105 | Dist_3.2 | 46.3% | 42.1% | 40.1% | 55.4% | 65.4% | 0.60 |
| 11 | Angle_105 | Dist_3.4 | 39.6% | 37.1% | 37.7% | 48.1% | 58.4% | 0.70 |
| 12 | Angle_105 | Dist_3.6 | 33.9% | 33.8% | 35.3% | 42.4% | 52.7% | 0.90 |
| 13 | Angle_105 | Dist_3.8 | 29.5% | 30.4% | 33.4% | 36.3% | 47.1% | 1.00 |
| 14 | Angle_105 | Dist_4.0 | 24.5% | 25.8% | 30.1% | 29.9% | 41.8% | 0.90 |
| 15 | Angle_105 | Dist_4.2 | 19.2% | 20.0% | 25.0% | 23.5% | 36.4% | 0.90 |
| 16 | Angle_105 | Dist_4.4 | 13.3% | 13.9% | 18.0% | 17.7% | 29.9% | 0.90 |
| 17 | Angle_120 | Dist_3.0 | 53.9% | 51.1% | 51.7% | 63.3% | 70.5% | 0.70 |
| 18 | Angle_120 | Dist_3.2 | 45.0% | 40.7% | 39.1% | 54.7% | 64.3% | 0.60 |
| 19 | Angle_120 | Dist_3.4 | 37.6% | 35.9% | 35.6% | 46.7% | 57.3% | 0.60 |
| 20 | Angle_120 | Dist_3.6 | 31.2% | 32.5% | 32.4% | 40.6% | 51.5% | 0.90 |
| 21 | Angle_120 | Dist_3.8 | 26.6% | 28.9% | 29.9% | 34.5% | 46.1% | 1.00 |
| 22 | Angle_120 | Dist_4.0 | 21.8% | 24.3% | 26.4% | 28.1% | 41.1% | 1.00 |
| 23 | Angle_120 | Dist_4.2 | 17.1% | 18.8% | 21.5% | 21.8% | 36.4% | 1.00 |
| 24 | Angle_120 | Dist_4.4 | 12.2% | 13.2% | 15.1% | 16.6% | 30.5% | 1.00 |
| 25 | Angle_135 | Dist_3.0 | 42.2% | 42.4% | 40.9% | 56.2% | 63.9% | 0.70 |
| 26 | Angle_135 | Dist_3.2 | 32.7% | 34.7% | 30.4% | 47.4% | 57.3% | 0.70 |
| 27 | Angle_135 | Dist_3.4 | 27.1% | 32.5% | 28.3% | 39.0% | 52.5% | 0.90 |
| 28 | Angle_135 | Dist_3.6 | 24.0% | 30.7% | 26.9% | 33.9% | 47.5% | 0.90 |
| 29 | Angle_135 | Dist_3.8 | 21.6% | 28.8% | 24.6% | 29.1% | 43.4% | 0.90 |
| 30 | Angle_135 | Dist_4.0 | 18.5% | 25.6% | 21.8% | 24.4% | 40.0% | 0.70 |
| 31 | Angle_135 | Dist_4.2 | 15.3% | 21.3% | 18.3% | 19.9% | 37.0% | 0.70 |
| 32 | Angle_135 | Dist_4.4 | 12.5% | 17.0% | 13.9% | 16.1% | 33.1% | 0.70 |
| 33 | Dist_3.4 | Angle_90 | 39.6% | 37.1% | 37.7% | 48.1% | 58.4% | 0.70 |
| 34 | Dist_3.4 | Angle_105 | 39.6% | 37.1% | 37.7% | 48.1% | 58.4% | 0.70 |
| 35 | Dist_3.4 | Angle_120 | 37.6% | 35.9% | 35.6% | 46.7% | 57.3% | 0.60 |
| 36 | Dist_3.4 | Angle_135 | 27.1% | 32.5% | 28.3% | 39.0% | 52.5% | 0.90 |
| 37 | Dist_3.6 | Angle_90 | 33.9% | 33.8% | 35.3% | 42.4% | 52.7% | 0.90 |
| 38 | Dist_3.6 | Angle_105 | 33.9% | 33.8% | 35.3% | 42.4% | 52.7% | 0.90 |
| 39 | Dist_3.6 | Angle_120 | 31.2% | 32.5% | 32.4% | 40.6% | 51.5% | 0.90 |
| 40 | Dist_3.6 | Angle_135 | 24.0% | 30.7% | 26.9% | 33.9% | 47.5% | 0.90 |
| 41 | Dist_3.8 | Angle_90 | 29.5% | 30.5% | 33.4% | 36.3% | 47.1% | 1.00 |
| 42 | Dist_3.8 | Angle_105 | 29.5% | 30.4% | 33.4% | 36.3% | 47.1% | 1.00 |
| 43 | Dist_3.8 | Angle_120 | 26.6% | 28.9% | 29.9% | 34.5% | 46.1% | 1.00 |
| 44 | Dist_3.8 | Angle_135 | 21.6% | 28.8% | 24.6% | 29.1% | 43.4% | 0.90 |
| 45 | Dist_4.0 | Angle_90 | 24.5% | 25.8% | 30.1% | 29.9% | 41.9% | 0.90 |
| 46 | Dist_4.0 | Angle_105 | 24.5% | 25.8% | 30.1% | 29.9% | 41.8% | 0.90 |
| 47 | Dist_4.0 | Angle_120 | 21.8% | 24.3% | 26.4% | 28.1% | 41.1% | 1.00 |
| 48 | Dist_4.0 | Angle_135 | 18.5% | 25.6% | 21.8% | 24.4% | 40.0% | 0.70 |
| 49 | Dist_4.2 | Angle_90 | 19.2% | 20.0% | 25.0% | 23.5% | 36.5% | 0.90 |
| 50 | Dist_4.2 | Angle_105 | 19.2% | 20.0% | 25.0% | 23.5% | 36.4% | 0.90 |
| 51 | Dist_4.2 | Angle_120 | 17.1% | 18.8% | 21.5% | 21.8% | 36.4% | 1.00 |
| 52 | Dist_4.2 | Angle_135 | 15.3% | 21.3% | 18.3% | 19.9% | 37.0% | 0.70 |
| 53 | Dist_4.4 | Angle_90 | 13.3% | 13.9% | 18.1% | 17.8% | 29.8% | 0.90 |
| 54 | Dist_4.4 | Angle_105 | 13.3% | 13.9% | 18.0% | 17.7% | 29.9% | 0.90 |
| 55 | Dist_4.4 | Angle_120 | 12.2% | 13.2% | 15.1% | 16.6% | 30.5% | 1.00 |
| 56 | Dist_4.4 | Angle_135 | 12.5% | 17.0% | 13.9% | 16.1% | 33.1% | 0.70 |

**Supplementary Table 1.** The inverse martensite selectivity of the system and the rate of p(C8only)/p(C7orC8) in the simulated trajectories of aMD at different angle thresholds.
